# Employees’ experiences of involving their managers in the Return-to-Work process through a three-party meeting in primary healthcare – a retrospective interview study

**DOI:** 10.1080/02813432.2025.2572123

**Published:** 2025-10-21

**Authors:** Marie-Louise Pauhlson, Teresia Nyman, Magnus Svartengren, Kristina Eliasson, Therese Hellman

**Affiliations:** ^a^ Department of Medical Sciences, Occupational and Environmental Medicine, Uppsala University, Uppsala, Sweden; ^b^ Occupational and Environmental Medicine, Uppsala University Hospital, Uppsala, Sweden

**Keywords:** Demand and Ability protocol, rehabilitation, three-party meeting, qualitative method, return to work, rehabilitation coordinator

## Abstract

**Purpose:**

A trusting relationship between employee and manager is crucial for constructive dialogue regarding work ability. However, employees may sometimes experience collaboration as unpleasant if the dialogue with their manager is not constructive. The aim of the study was to explore how employees on sick leave experience manager involvement in the RTW through a three-party meeting using the Demand and Ability Protocol (DAP) in primary healthcare.

**Materials and methods:**

Data included 20 semi-structured individual interviews with employees diagnosed with common mental disorders or musculoskeletal disorders who had participated in a DAP dialogue. Thematic analysis was used to analyse the data.

**Results:**

Employees wanted to reach out to their manager but had challenges getting the message through about their reduced work ability. During sick leave, the structured DAP held within primary healthcare was experienced as a helpful measure to foster collaboration with the manager. The dialogue helped explore the balance between workplace demands and the employee’s capabilities. This enabled both parties to share their view and the rehabilitation coordinator could guide towards potential adaptations. Employees found that the increased mutual understanding fostered by the DAP helped pave the way for ongoing collaboration in the RTW process.

**Conclusions:**

The results underscore the importance of facilitating collaboration between employees and managers before, during, and after sick leave. The DAP can support the development of a trust-based relationship that enables all involved stakeholders to articulate needs, propose measures, and make informed decisions that enhance efforts throughout the RTW process.

## Introduction

In the past few years, the number of sick leave cases in Sweden has increased, where the major diagnoses causing sick leave rates are common mental disorders (CMD) and musculoskeletal disorders (MSD) [1]. These diagnoses also account for a large part of work disability worldwide, making sick leave and absence from work a considerable social and economic burden, not only for employees but also for managers and society at large [2–4].

Work has a great impact on the health and well-being of individuals [5]. Initiating and promoting Return-to-Work (RTW) is, therefore, one of the most important goals in the rehabilitation process. The increasing pace and complexity of demands in contemporary working life require effective collaboration among all stakeholders involved: the employee, manager, and occupational and healthcare professionals, among others [6]. Each stakeholder has a specific role and responsibility, depending on their tasks and mandates. Primary healthcare is the arena that cares for people with CMD and MSD, with an important contribution to RTW [7–11]. Since 2020, Swedish legislation has ensured that individuals on sick leave are entitled to support if there is a need with the coordination of rehabilitation within primary healthcare. This aims to facilitate both internal and external collaboration between the stakeholders without taking sides in order to identify appropriate measures for the RTW [12]. Facilitation of collaboration can be performed by a rehabilitation coordinator and research shows that the support can positively influence the duration of sick leave and provide benefits in the RTW process [13]. However, opposite conditions also occur [14] or showing no significant difference regarding reduced sick leave or speed of RTW [15,16]. Furthermore, the rehabilitation coordinator has been found to enhance supportive collaboration between the employee, manager, and other stakeholders, playing a crucial role in the RTW process [17,18].

The obligations of the employer are of considerable importance according to the EU Occupational Health and Safety (EU-OHS) legislative [19]. In Sweden, employers have a far-reaching responsibility for the vocational rehabilitation of employees on sick leave and for ensuring sustainable working conditions overall [2,20]. The responsibility for the practical implications of rehabilitation is usually delegated from the employer to the employee’s immediate responsible manager. Accordingly, employees are expected to actively participate and communicate their needs for workplace adaptations in dialogue with their manager when necessary [20]. Even though the legal framework regulates collaboration in the RTW process, it may still be difficult for employees to voice their concerns and engage with their manager. Revealing limitations in one’s work ability, where an employee’s abilities cannot meet the demands of the work and expressing the need for workplace adaptations during the RTW process, can place the employee in a vulnerable position [21,22].

A trusting relationship between employee and manager has been shown to be crucial when discussing changes in the employee’s work ability [23]. However, employees may sometimes experience collaboration as unpleasant if the dialogue with their manager is not constructive [22,24]. One approach to foster collaboration is through work-oriented three-party meetings, involving the employee and manager in a structured dialogue facilitated by a healthcare professional. Using a dialogue tool can further support discussions about work tasks and the employee’s current work ability, aiming to identify suitable, personalised adaptations that facilitate the employee’s RTW process. Such tools are available, developed specifically for employees on sick leave due to particular diagnoses, such as CMD, stress-induced exhaustion disorder (SED) and MSD [25–27]. These tools aim to enhance collaboration between employees and managers, and studies have shown that they can promote and improve the RTW process, by providing a formal structure that facilitates cooperation and makes room for communication about pain and mental health in relation to work ability [28–31].

The Demand and Ability Protocol, DAP, is one dialogue tool based on a three-party meeting, targeted at facilitating the collaboration between the employee and manager in the RTW process [32]. This tool involves a structured assessment of the balance between the demands of the employee’s current work and their functional abilities. Any identified imbalances, together with possible adaptations and measures, are then summarised in an action plan that aims at progressing the RTW process. The DAP is distinctive as a generic tool as it is theoretically designed for use regardless of diagnosis or occupation [32]. The generic use of the DAP has empirical evidence hence it has been tested in specialised pain rehabilitation with patients representing various occupations and the findings showed that its clear structure was appreciated and found helpful by users [33]. Furthermore, it has been piloted within the occupational healthcare context with patients having quite similar work situations but various diagnoses and limitations, and findings showed that it can facilitate the formulation of workplace adaptations, when necessary [34]. The DAP has also been tested for content validity, where the results confirmed that the DAP is a relevant dialogue tool to support collaboration among employees, managers, and facilitators in health and occupational healthcare when discussing work ability [35]. It provides extensive user options, as it based on empirical use can be used preventively, during sick-leave and as a follow-up measure, enhancing collaboration between the employee and manager [32]. There is currently no evaluation of employees’ experiences of using the DAP in a three-party meeting within a primary healthcare setting.

## Aim

The aim was to explore how employees on sick leave experience their manager’s involvement in the Return-to-Work process through a three-party meeting using the Demand and Ability Protocol (DAP) in primary healthcare.

## Materials and method

### Design

A qualitative, inductive and retrospective approach using semi-structured individual interviews was employed [36,37] to explore how employees on sick leave experience their manager’s involvement in the Return-to-Work process through structured three-party meetings using the DAP. The study is reported in accordance with the Consolidated Criteria for Reporting Qualitative Research (COREQ) guidelines [38].

### Ethical considerations

The study was approved by the Ethics Review Authority (identification number 2021-01577 and 2021-06950-02) and conducted in accordance with the Declaration of Helsinki [39]. All participants received both oral and written information prior to the interviews, with particular emphasis on the fact that participation was voluntary. They provided written informed consent, which was de-identified and assigned a code to ensure confidentiality.

The data were handled in a safe manner and stored according to the regulations of Uppsala University regarding the collection and storage of research materials. The analysis was carried out on pseudonymized data as each participant was given a unique code number. The results were presented in a way that ensured no individual included in the study could be identified.

### Participants and recruitment

The recruitment of participants took place in two regions in the middle of Sweden in collaboration with five rehabilitation coordinators at primary healthcare centres. In recent years Swedish healthcare has had a legal responsibility to provide coordination services to patients at risk of becoming sick-listed, or to those on sickness absence [12]. The coordination service is often provided by a rehabilitation coordinator and includes: 1) personal support for patients, 2) managing internal collaboration within the health care services, and 3) external collaboration with, for example the patient’s employer [12]. The rehabilitation coordinators participated in a half-day course in the DAP to apply it uniformly in the three-party meetings. Training in performing the DAP was provided by the first and last author.

Consecutive sampling [40] was used to select eligible participants who were employees with CMD or long-term pain, for whom the rehabilitation coordinator were planning for a three-party meeting using the DAP and involving the employer. Participants were required to be on ongoing sick leave, either full-time for a maximum of 12 months or part-time for a longer period (up to 24 months). Further inclusion criteria were an age range of 18 to 65 years. Exclusion criterion was having multiple diseases.

The study included 20 employees, sixteen women and four men, with an average age of 42 years (23–59 years), working in occupations in health- and social care, childcare, education, retail, construction, and a few as clerical staff or officers. Five employees lived in the city, the remaining lived in rural settings. The majority were on sick leave fulltime at the time for recruitment and had been so for approximately 3–9 months, due to CMD (*n* = 18) or long-term pain (*n* = 2). None of the employees had any previous experience of three-party meetings using the DAP.

The employees received verbal and written information from the rehabilitation coordinator regarding the purpose of the study and what participation entailed when a three-party meeting involving the DAP was planned. If an employee was interested in participating and wanted more information, the rehabilitation coordinator gave their contact information to the first author, who, in turn, contacted the employee by phone to provide more comprehensive information about what participation entailed. Information was provided about the possibility of cancelling the study participation at any time without needing to give a reason. If the employee agreed to participate, an informed consent form was sent by mail to be signed and returned to the first author using a pre-paid envelope. All participants who received information about the study by the first author agreed to participate. However, we do not have information regarding how many patients the rehabilitation coordinators informed.

During a second phone call, once the signed consent had been received, a time was arranged for a research interview, within a month from the date of the three-party meeting. The employee decided when, where and how the interview would be conducted to ensure a sense of security and trust during the interview situation [41].

### Data collection method

The interviews were conducted between June 2022 and April 2023 and lasted between 20 and 60 min. Three interviews were held face-to-face at one primary healthcare centre and the remaining interviews were conducted *via* video meetings in Zoom Conference System. All interviews were carried out about a month after the three-party meeting and the use of DAP dialogue.

A semi-structured interview guide was designed according to the guidelines set by Patton [37] and Kvale and Brinkmann [42]. This guide ensured that the interviews were carried out in a similar manner, with all participants being asked questions around key themes (see Supplementary material: Interview guide for the interview guide with probes). These themes covered background information about the employee, their work situation and rehabilitation before the DAP took place, their experience of the three-party meeting, and the perceived contribution of the meeting to their rehabilitation process and planning of RTW.

The research interviews were conducted by the first author, who had no involvement in the employees’ RTW process, to avoid any dependency. The first author has extensive experience conducting clinical interviews from working as a physiotherapist. A pilot interview was initially conducted to adjust the questions in the interview guide if necessary and to assess the time required [37,43]. Minor reformulations were made, but these did not affect the content of the interview guide as the structure was kept intact. The pilot interview was also included in the study. At the start of each interview, the employee was informed both verbally and in writing about confidentiality, anonymity and the fact that no personal information could be identified [37]. The interviews were recorded with a dictaphone or the computer’s audio recording function, without video capture, ensuring that the interviewer could focus fully on the employee without needing to take notes at the same time [37]. The total number of interviews was set at 20, as this was deemed sufficient to generate enough information power to answer the study’s aim [44].

### Data analysis and coding process

All interviews were transcribed verbatim using Kahubi – Avidnote (autumn 2024 version), a transcription software programme. Thereafter, the interviews were compared with the original recordings and double-checked by the first author, who corrected any obvious typos.

The material was analysed using thematic analysis inspired by Braun and Clarke, applying an inductive approach. This method was considered suitable as it aims to explore experiences to understand participants’ processes and perspectives [45,46]. The first step of the analysis involved getting to know the material by organising and outlining the collected data [45]. All interviews were listened to again by the first author and compared with the transcription. The material was then reviewed on repeated occasions to deepen understanding and gain an overview of the contextual content [45].

In the second step, a basic thematic coding of one interview was carried out by the first author and subsequently compared with the coding of the same interview by the last author. A discussion followed about how the coding had been carried out, as well as a comparison of identified basic units. As the basic coding was found to be consistent between the first author and the last author, the first author proceeded to code all remaining transcripts in the same way. The procedure was verified by the last author regularly to enhance credibility. For each interview, individual answers were compiled to obtain a coherent and clear summary. Memos were taken throughout the entire procedure, and in cases of differing points of view between the researchers, discussions were held to reach a consensus.

In the next step, the basic data units from all interviews were interpreted, categorised and organised into preliminary themes. A mind mapping approach was used to collect and illustrate the different meaningful units, providing a clear and concise overview of the material. This process involved going back and forth to find and combine the generated initial codes, ensuring that the entire spectrum of the employees’ experiences was captured. Further discussion and dialogue between the researchers followed this step to compare interpretations and confirm overall consistency in the coding.

Thereafter, the process of identifying themes commenced, during which the researchers decided whether to retain or modify any of the generated themes. Through discussions within the entire research group, the themes were further defined and named. In the next step, the group reviewed and refined the themes, which resulted in additional subthemes. Finally, a last round of analysis was carried out to ensure that the rich and nuanced data had been thoroughly captured and gathered.

To provide examples and link them to the interviews, quotations from the employees are used to support the statements. Out of 20 employees, quotes of 10 are presented in the results. As themes reoccurred during the analysis, the research group thoroughly discussed and considered the chosen quotes to be representative of the entire group of employee’s experiences.

### Research group and reflexivity

As the research group is multi-professional, consisting of a physician, an occupational therapist, and three registered physiotherapists specialising in ergonomics, all with extensive knowledge of the setting and experience working in RTW processes, it must be considered that this may influence how the material is approached and interpreted.

To ensure that this pre-understanding did not affect the results to any possible extent, instead contributing to the perspectives, it was of utmost importance to create space for ongoing discussion about the interpretation and framing of the material throughout the entire analysis. In this way, the reflexivity of the study was strengthened, thereby enhancing the overall quality of the research [47,48]. Using interviews as a method for collecting qualitative data is a common and well-established approach. While interviews offer many benefits for capturing employees’ experiences in-depth, it is important to remember that, during the interview process, there may be reasons to be observant of power relations. This could be the case, for instance, if the researcher holds a higher level of education than the participant, who may also be in a vulnerable situation due to being on sick leave [41]. Furthermore, in this study, all employees had voluntarily agreed to participate, suggesting that they were able to decide for themselves what they wanted to share regarding their experiences.

## Results

**Figure 1.**
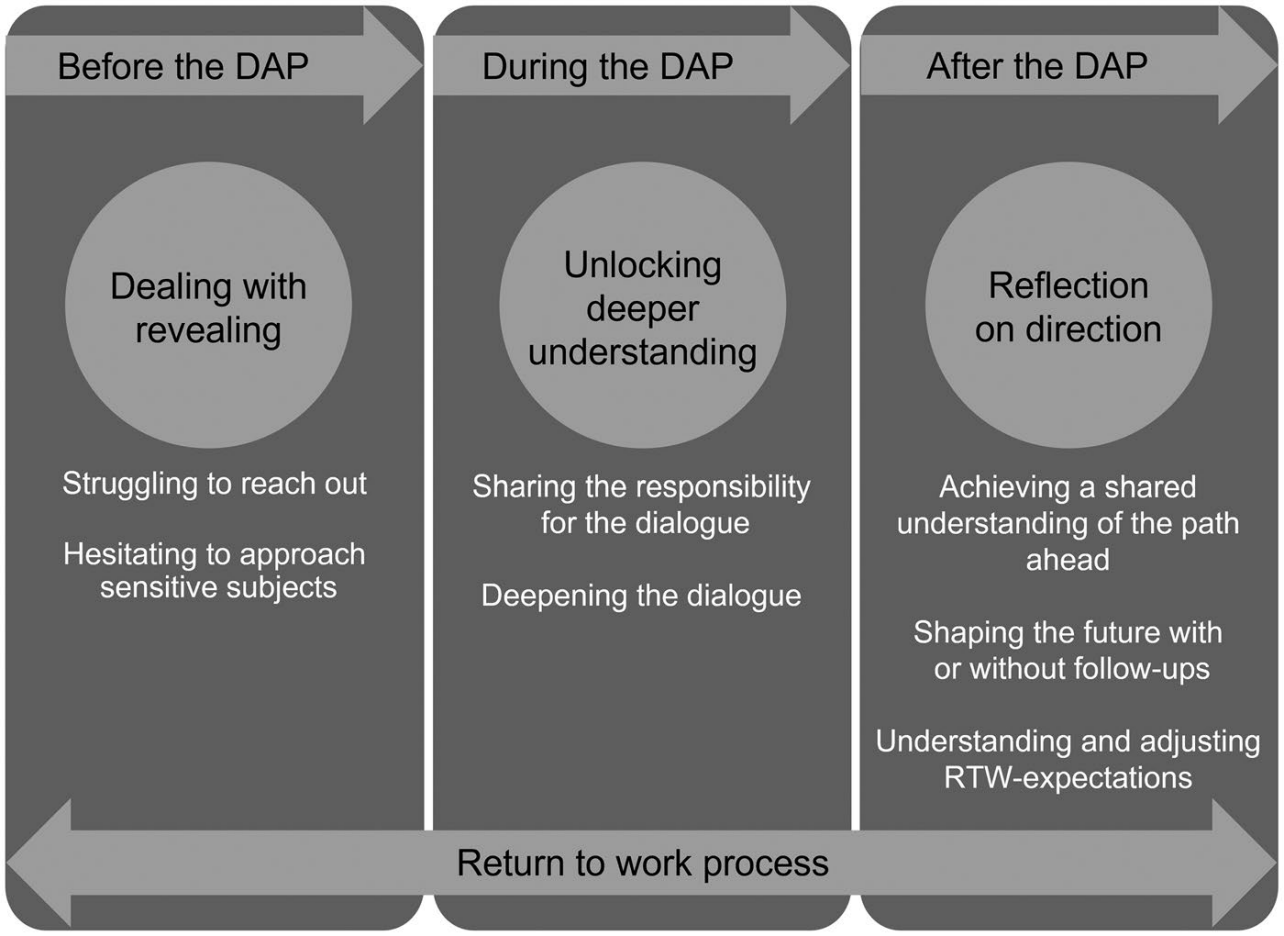
Illustration of the three themes with Sub-themes, linked to the timeline of the DAP.

The qualitative analysis identified three main themes: (1) Dealing with revealing, (2) Unlocking deeper understanding through structured dialogue and (3) Reflection on direction. The analyses also resulted in seven sub-themes. An overview of themes and sub-themes is presented in Figure 1.

### Theme 1. Dealing with revealing

Most employees described that before going on sick leave, they had tried to talk to their manager in different ways to raise awareness about the strained work situation and how this affected their health and well-being in a negative way. There were, however, circumstances that made it difficult for them to be as straightforward as needed in these communications.

#### Struggling to reach out

Although many employees experienced that they had a good relationship with their manager, some felt they were not taken seriously when raising difficult issues or concerns about a stressful work environment.

I flagged it for a long time during the spring; that things weren’t going well with us. But I don’t think I really got through… and it can be like that when you keep flagging it up and saying hello, hello, listen. But there’s hardly ever time to listen, or no one wants to listen. (Employee 8).

Others expressed difficulties in communicating with their manager as they did not have a close relationship. In some cases, this was a result of frequent changes in managers coming and going within a relatively short period. Although this was not a big concern for the employees, they did express that the consequence was simply having no manager available whom they could trust and approach naturally.

When I started there, I had one manager. Then he left, and we got a new one… Then we got yet another manager. And she left, so we got another one […] And then we got the fifth manager, who has been around the whole time I’ve been off sick. And she has been really good. But then she left too. So now we have a manager starting here. I’m going to check today when she’s due to start. (Employee 7).

Several employees also described other reasons that made it difficult to reach out to their manager. It could be the case of the manager having the main office far from the employee’s workplace, which made it difficult to just drop by for a quick chat. In those cases, it was often emphasised that the manager was only a phone call or message away, but employees could still find it an insurmountable barrier to pick up the phone, especially if the employee was stressed or had health issues. The employee could also perceive that the manager had low attendance at the workplace, which was interpreted by the employee as being due to the manager’s heavy workload. This could lead the employee to feel that there was little room to raise their concerns, not wanting to add to the manager’s burden, as they already seemed to be under considerable strain.

#### Hesitating to approach sensitive subjects

No matter how good a relationship an employee had with the manager, the experience was present among many of the employees that conversations concerning their health and ability to work while on sick leave were challenging and sensitive.

I was ashamed of being on sick leave and didn’t want to talk to my managers. I think it took some time before we actually spoke—right from when I called in sick. Of course, I had to keep them updated about how the sick leave was going, but it was a bit sensitive in the beginning, I think, from both sides. I felt a kind of shame. I had never experienced anything like this before and felt embarrassed about where I had ended up. So, I found it very difficult to talk about it. (Employee 12).

The feeling of doubting their own capability and not contributing enough as an employee, together with the experience of being left alone without support, made it even more difficult to speak openly with the manager. As an employee, one does not want to appear weak, while reduced work ability is generally not viewed as an asset in the workplace and brings no advantage – in fact, quite the opposite. The employees felt uncertain about their own work ability in relation to the demands at work. Nevertheless, there was a strong willingness among them to express their feelings and needs, often because it was clear to them that the manager did not have the full picture of their situation at work. In some cases, employees realised that their managers had completely incorrect information about their health and sick leave; consequently, the employee felt an urge to explain their circumstances, hoping for understanding.

The manager didn’t have the full picture. His interpretation was probably that everything was fine and there had been no problems at the workplace. (Employee 5).

### Theme 2. Unlocking deeper understanding through the structured dialogue

#### Sharing responsibility for the dialogue

When employees on sick leave received information from the rehabilitation coordinator in primary healthcare about the DAP and that the dialogue tool could be used together with the manager when discussing ways to RTW, the majority regarded it as a good intervention.

I don’t remember it entirely, but it was about going through a number of points to identify what I need, what I struggle with, and what I am capable of. Yes, I thought I’d accept all the help I could get. (Employee 16).

A few were cautiously negative at first, as they did not want to end up in a disagreement with their manager, something they felt unequipped to handle. However, after receiving further information about the DAP as a three-party meeting where the rehabilitation coordinator was the facilitator, they changed their mind.

So, there was some worry about meeting my manager, who perhaps wouldn’t fully understand the situation. But when she—the rehabilitation coordinator—explained that there is a very clear tool to keep us on track, it felt reassuring to know there was a structure in place. (Employee 1).

The employees felt relieved to share the responsibility of managing the conversation and discussing possible adaptations and solutions for the RTW process together with the manager, under the guidance of the rehabilitation coordinator.

#### Deepening the dialogue

The employees considered the DAP as concrete, structured and clarifying. By following the form of the conversation, the DAP helped to keep the three-party meeting focused and prevented it from drifting off-topic. In addition, the employees experienced that the DAP allowed everyone to express their viewpoints regarding the demands of the work. A few mentioned the DAP having too many questions and therefore took time to implement. This was something that took its toll on the employees. Still, most of the employees pointed out that this approach ensured that no important aspects of the work tasks or work situation were overlooked.

It was probably the first time I felt we stuck to the subject and didn’t just drift off. Previously, we had been in rehab meetings, but nothing was ever really decided properly. (Employee 17).

The employees expressed that discussing their functional ability so openly, as they were allowed to do, thanks to the structure of the DAP, felt somewhat contradictory. It was not something they were used to, and in many ways, it was a mixed experience, evoking conflicting feelings.

No, well, it felt a bit difficult, but at the same time it was a relief, and difficult in the sense that it became so clear that… yes, something is wrong. What is it? That’s how it felt. But at the same time, it was a relief that it was clarified. (Employee 6).

Two employees said that the feelings of unpleasantness remained during the DAP and one expressed the DAP having no strengthening influence at all relationwise the manager. Still, it encouraged many employees to stand up for themselves, their functional capacity and their needs, something they had often previously felt unable to do. The structure of the DAP was felt to facilitate the dialogue with the manager, even in cases where the employee experienced that the manager tended to downplay the demands of the work and the work environment.

The employees felt free to speak because they perceived the rehabilitation coordinator as a professional and neutral facilitator who supported the conversation during the DAP and created a safe situation. Additionally, the rehabilitation coordinator made suggestions for work adaptations, and several employees pointed out that it was not a matter of the rehabilitation coordinator taking sides but acting as a mediator, something the employees felt strengthened the collaboration throughout the DAP.

### Theme 3. Reflection on direction

#### Achieving a shared understanding of the path ahead

The common experience among the employees after completing the DAP was that it helped their manager to gain a greater understanding of their work situation and health, as well as what might be needed to facilitate their RTW. This also made the employees feel less alone and more confident that there really was a path back to work.

The relationship with my manager improved a little after the rehabilitation coordinator’s involvement… after that, she also became much better at following up. And I had no expectations of her before. I gained trust, like I understood… It also strengthened the feeling that maybe this time it could actually work, almost just focusing on keeping things positive. (Employee 8).

Furthermore, the collaboration through the DAP provided the employee a deeper understanding that, in many work situations, the higher demands were often self-imposed rather than coming from the manager’s point of view. Being able to share and discuss this during the three-party meeting led to the manager adjusting their expectations regarding work demands, which, in turn, helped the employee develop a more accepting view of their own work ability, thereby building trust in the collaboration with the manager.

Yes, I got a completely different perspective there and felt that, okay… we should check in on how much needs to be done so I don’t have to feel this stress. That felt really good. I want to take on this responsibility and manage it. Yes, I’ll sort it out, but now…(Employee 12).

When the DAP was implemented, a way forward emerged, which could take different forms for the employees. The more detailed and structured these measures were, the clearer the employees felt that the path to RTW became. Another experience described was that the DAP helped to visualise the work situation itself as both challenging and difficult.

My job is difficult, and that became very clear during the DAP. I think it just became really obvious… The job demands so much from you. It was clear that it wasn’t me who was incapable. Honestly. There was also a small list of adjustments I could have… and that felt good. (Employee 8).

By illustrating this through the DAP and having it confirmed by the manager, employees could experience a sense of validation, which, in turn, could contribute to increasing their motivation to RTW. Before this had been clarified in the DAP, some employees felt that discussions about RTW focused primarily on getting them back to work as smoothly and quickly as possible to address accumulated tasks at the workplace, with adaptations being somewhat overlooked in the dialogue with the manager.

Through the DAP, it became possible for many employees, together with the manager and with the guidance of the rehabilitation coordinator, to appropriately tailor possible adaptations and develop a realistic plan moving ahead. The adaptations and actions agreed upon for the RTW were also written down on paper. Having a signed action plan was experienced by the employees as a formal agreement, which they perceived as reasonable to pursue.

#### Shaping the future RTW process - with or without follow-ups

About half of the employees in the study described that they had a clear plan for how the follow-ups of the RTW would take place, together with their manager, and sometimes also with the rehabilitation coordinator. These employees expressed satisfaction with this planning, as it gave them considerable hope for a sustainable RTW through the structured plan for the follow-up meetings.

But it was an initiative from my employer (with the follow-ups). A way of showing that they want to do their best to help me return. And that really meant that I could plan my return in a different way. Truly! Because I had that support… It gave me the opportunity to gradually work my way back. (Employee 1).

Employees who did not have a clear plan for follow-ups, either with their manager or the rehabilitation coordinator, had different opinions about this arrangement. Some expressed strong confidence in the understanding that they could contact their manager whenever needed or get in touch with the rehabilitation coordinator to make an appointment. They expressed great confidence that this support existed, just a phone call or e-mail away.

She will be able to meet all my expectations. She will be supportive and be there when I need her. Or if she can’t talk or have a conversation right now, she makes sure to schedule one as soon as possible. Yes, if she’s not available in person. (Employee 11).

Others reported feelings of dissatisfaction and uncertainty about the fact that there was no planned follow-up, describing this as a recurring pattern they had experienced before in their employment. They felt left to their own devices and, once again, found themselves in a vulnerable position. The absence of a structured follow-up process for the RTW made the employees feel less motivated to get back to work, as there were no clearly planned steps to support their RTW.

#### Understanding and adjusting RTW- expectations

Taking part in the DAP encouraged employees to look ahead and make plans, as the conditions for RTW emerged during the collaborative dialogue in the three-party meeting. When the employee discussed their functional ability and the demands at work with the manager during the DAP, it highlighted factors that needed to be considered in the RTW process. One example was the recognition that the RTW needed to be gradual and take its time. Speeding up the RTW was not a priority if the imbalance between the employee’s ability to perform work tasks and the demands of the job was clearly highlighted through the DAP. In many cases, adaptations could be made, but sometimes it also became apparent that it was not possible to return to the specific work tasks until the employee’s functional capacity had improved.

It has to be allowed to take time to return to work, and my manager understands that. That it needs to take that time, really. You can’t fix everything at once. But just because it’s like this now, doesn’t mean it will always be this way. No, that’s how it is. But you do feel that it’s going so slowly. (Employee 10).

Even though it had not been easily achieved, the employee described having gained a deeper understanding of their own functional capacity and needs during the sick leave period, which could be confirmed through the use of the DAP. The structured dialogue helped the employee to express the necessity of a step-by-step RTW and to ask the manager for support in ensuring a steady pace when going back to work. Being in this situation placed the employee in a vulnerable position, often accompanied by stress and frustration over not being able to recover quickly enough. However, the transparent collaboration facilitated by the DAP helped create the conditions for a continued RTW based on mutual understanding. Ultimately, the challenge for the employees was still to manage the RTW in collaboration with their manager in such a sustainable way over time, so that setbacks could be avoided.

I’m used to having complete control over everything, all the time—both at home and at work. I have to hold myself back so I don’t get too involved. So this is something I need to work on for my own sake. I need to let go of certain things and simply accept that this is how it is right now. But at the same time, it’s also that drive I have—for things to work. (Employee 11).

## Discussion

The findings of the study reveal that the employees on sick leave generally had a positive experience of their manager’s involvement in the RTW process, including participation in a three-party meeting. The majority of the employees found that the structured DAP dialogue strengthened their RTW by enhancing joint efforts and supporting continued collaboration.

Employees wanted their manager to be involved and active in the RTW process, before, during and after the DAP. There was a strong desire among employees to be able to easily express their concerns regarding declining health and/or a stressful work situation, even before sick leave became relevant. Communicating personal needs and potential adaptations with the manager is regarded by employees as an important part of effective collaboration, as shown in previous studies [49–53]. Nevertheless, from the employees’ point of view, conveying this message remained a challenge. When the manager was not sufficiently present at the workplace to allow for natural day-to-day collaboration, it reduced the opportunities for a dialogue on an everyday basis. Furthermore, employees often found it difficult to disclose a reduced ability to work, as talking to the manager about personal weaknesses or limitations in their work ability is not necessarily considered as part of everyday communication with a manager [54]. Often, this was combined with the feeling of unease about not contributing at work according to their own expectations. However, in some cases, the employee’s own expectations of work ability were higher than those held by the manager [33]. Taking advantage of the opportunity to use DAP as a structured dialogue tool as soon as possible when concerns about work ability arise can help reduce negative experiences and make the collaboration less tense.

One benefit of the structured review of work demands provided by the DAP was that it helped employees talk about health and stress-related issues without feeling like they were portraying themselves negatively. This has previously been highlighted as an advantage of using the DAP in the RTW process [33]. The DAP could highlight imbalances between the expectations of employees and managers and help them to come to speaking terms in this situation, something employees experienced as promoting the RTW process.

The employees in the study also expressed that they wanted the RTW collaboration to be objective and transparent. The majority of employees stated that with the structured format of the DAP, this clarity made the manager’s involvement more straightforward and facilitated collaboration considerably. This contrasted with their previous meetings with the employer before and during sick leave, which had tended to focus more diffusely on the employee’s current well-being and general health. Being able to plan for the future in terms of RTW has been shown to provide a sense of security for both the employee and the manager [33,34]. Overall, the employees perceived that the DAP dialogue strengthened their relationship with the manager, not only during the three-party meeting but also going forward, helping to lay the foundation for continued collaboration. These findings suggest that the DAP is effective across different patient groups, at different stages of sick leave, and in different healthcare contexts.

Most of the employees in the study described that, through the DAP, it became possible to plan for appropriate adaptations in the RTW process that were truly tailored to their individual needs. Together with the manager, they were able to discuss how to implement these adjustments in the workplace. The rehabilitation coordinator contributed specific knowledge about RTW processes and workplace adaptations during the three-party meeting and took into consideration applicable regulations and legislation, helping to bridge the gap between healthcare and the workplace [18,33,55]. The adaptations were documented on paper by the rehabilitation coordinator in the action plan as a summary of the DAP during the three-party meeting and signed by both the employee and the manager. Having a signed action plan was perceived by the employees a formal agreement, which was considered to be of importance for continued collaboration with the manager and for supporting the RTW process. In a previous study, the action plan was found to be helpful when implementing the planned adaptations in practice, serving as a helpful tool for turning intentions into concrete actions in the workplace [33].

When writing and signing the action plan after the DAP had been completed, it also became apparent that the employees considered planning for the follow-up of the RTW to be just as important as identifying the right adaptations. Even if it had not been easily achieved, the employees expressed that they had gained a deeper understanding of their own abilities and needs during the sick leave period. In many cases, this enriched self-knowledge could be linked to the demands of their work and validated by using the DAP. This helped the employee to clarify the legitimate reasons for how and when to follow up on the adaptations that needed to be addressed, and to emphasise the importance of consolidating these within the RTW process. The employee also felt that this made it possible to involve the manager in providing support and ensuring a steady pace when going back to work, a collaboration that has been found to be of importance in previous studies [24,33]. However, employees’ conditions for RTW are not static and as these may change over time, employees emphasised the importance of promoting ongoing collaboration between the employee and manager, with the past in mind, the present at hand, but with a focus on the future. This could be facilitated through follow-ups of the RTW process. Employees had varying experiences of both regular and non-existent follow-ups after the DAP. Although creating conditions for an employee to function at work has been found to be central to effective collaboration, research shows that this is not always the case [18,56]. Future studies are needed to develop knowledge on how structured follow-ups after completing the DAP can be incorporated into its use during the RTW process. Furthermore, it would be valuable to explore the managers involvement in the RTW process in a prospective study.

Even though today’s working life turns towards a greater understanding and acceptance of variations in work ability during the span of a working life, certain dilemmas remain that need to be addressed. From our study, it became evident that employees on sick leave often do not want to stand out at the workplace or feel like a burden to their employer. We argue that changes in an employee’s work ability over time should be considered as a natural topic for collaboration with the manager, being part of regular check-ups at work, whether on a day-to-day basis or at regular intervals. Recognising the value of ongoing collaboration between the employee and manager, as an obvious part of the work, and preferably in a proactive manner, may help foster open dialogue about ill health, work-related strain and imbalances in work tasks at an early stage. This, in turn, could help to prevent sick leave and promote sustainable RTW.

## Strengths and limitations

The important strength of this study is its exploration of employees’ experiences participating in a three-party meeting using the DAP in collaboration with their manager and a rehabilitation coordinator within primary healthcare.

Previous qualitative research has shown that participants can experience interviews as a valuable opportunity for reflection [37]. However, there is always a risk of misinterpreting the interview material, known as confirmation bias [37]. The researcher who conducted the interviews in the present study was not involved in delivering the DAP or in the employees’ treatment. This helped minimise the likelihood that participants felt compelled to report only positive experiences due to dependence on the primary healthcare staff they had contact with [37,54]. The fact that the interviews were conducted by someone outside the treating rehabilitation team can be said to strengthen the credibility of the study, helping to avoid potential power relations [48].

The interviews were conducted approximately one month after the three-party meeting using the DAP. Employees were still able to remember the conversation clearly, thereby eliminating the risk of recall bias [37], which can occur if participants do not remember past events correctly or omit details when reporting them [44]. The study population consisted of four men and sixteen women, working in health-, social- and childcare, education, retail, construction, with a few clerical officers. Although the sample lacked variety, it reflected the typical demographic of people on sick leave due to mental illness and pain in society [7,29,30].

While the rehabilitation coordinators informed eligible participants about the study, the research group does not have the full insight in the number of informed employees. This might be a potential risk to selection bias. However, the employees were included in the study before they participated in the DAP dialogue. This minimized the risk to only include employees with positive experiences which is reflected in the findings that also include negative experiences of both collaboration with manager and the DAP dialogue.

Out of 20 interviews, 17 were conducted digitally *via* video meetings in Zoom Conference System, a choice made by the employee. It is important to consider both the strengths and challenges of digital interviews as well as with in-person interviews [43]. However, digital video meetings can be a good alternative to capture experiences of employees that otherwise would not consider or be able to participate [43].

Since qualitative research involves the researcher as a key tool, it is also important to reflect on one’s pre-understanding to minimise various biases [37]. The interviewer has previous experience of the DAP which could lead to positive bias in the interview situation. To reduce this, it is crucial to maintain objectivity and be self-aware of own preconceptions. In this study this meant to carefully follow the interview guide and to use peer debriefing in the research group to share interpretations to confirm accuracy and ensure the interviewer is not projecting own views. To continuously return to the empirical material in an iterative process during the whole analysis procedure is yet another way. There is a risk that thematic analysis may be influenced by the researcher’s theoretical interest in the subject. To mitigate this, the material should be analysed by at least two researchers, which strengthens the reliability of the analysis by reducing the likelihood that an individual researcher’s pre-understandings colour the results [37]. Furthermore, writing the text should be an integral part of the entire analytical process, beginning with the first stage of identifying and reviewing ideas and codes [37,44,57].

## Conclusion

An overall finding was that employees on sick leave in primary healthcare had a positive experience of their manager’s involvement in their RTW process. The structured three-party meeting using the DAP strengthened their RTW by enhancing joint efforts for continued collaboration. While changes in an employee’s work ability over time are to be expected, recognising this as a natural topic for collaboration with the manager could be beneficial. Proactive collaboration that facilitates early discussions about ill health, work strain and imbalances in work tasks may help prevent sick leave and using the DAP as the dialogue tool can help promote sustainable RTW.

## Supplementary Material

Supplemental Material
