# Supplementary material for: Employees’ experiences of involving their managers in the Return-to-Work process through a three-party meeting in primary healthcare – a retrospective interview study
Source: Scand J Prim Health Care. 2025 Oct 21;44(1):1–15. doi: 10.1080/02813432.2025.2572123 (PMC12918362; doi:10.1080/02813432.2025.2572123)
Supplement: Supplemental Material [file IPRI_A_2572123_SM3805.docx]

**Supplementary material**

Interview guide with examples of probes and follow-up questions.

**INTERVIEW GUIDE PATIENTS**

*Respondent introduces himself/herself*

How long have you had contact with the rehabilitation coordinator?
How long and to what extent have you been on sick leave?
If you think back to the past year – to what extent have you worked? Repeated short-term sick leave? Full-time absence? Part-time absence?

If you have been on full-time sick leave, have you had contact with your work during that period?
- Manager? Colleagues? HR?

How long have you been at your current job?
What is your job title/occupational title?
Have you had any contact with your occupational health?

**Before the DAP dialogue**

What did you think about your work before rehabilitation?
Were you able to influence the work situation?
*For example: physical demands, pace, quality, deadline, attendance, own responsibility*

Were you motivated to work? Did you enjoy your work?
How was the dialogue/relationship with your manager?
How was the dialogue/relationship with colleagues?

The work situation onwards…
*For example: increase in time, continued sick leave, when returning to work may be appropriate, changing jobs/staying put in place*

What did you think when you were offered a three-party meeting with your manager?
How was the three-party meeting presented to you by the rehabilitation coordinator?
What expectations did you have for the three-party meeting and the DAP?
Was the purpose of the three-party meeting and the DAP clear to you? What did you think about it?

**The DAP and three-party meeting**

How did you experience the DAP and three-party meeting?
Can you describe something that felt really good? (Preferably concrete examples)
Can you describe something that felt less good? (Preferably concrete examples)
Was something missing?

How did you experience discussing your functional ability with your manager?
Was the format of the conversation supportive, or on the contrary, did the format make it more difficult to talk about your functional ability?

Did you and your manager reach a consensus on the work requirements?
If you disagreed, how did you handle it?
How did you reach a consensus on the work requirements?
Did everyone get a chance to speak?

How did you experience the rehabilitation coordinator influencing the conversation?
How did it feel to have the rehabilitation coordinator involved?
Was the role of the rehabilitation coordinator clear?

In what way did your view of work change after the DAP and three-party meeting?
*Motivation to return*
*Possibility for adaptations*
*Attitude of manager*

If it had not changed; would you have liked the three-party meeting and the DAP to lead to a change? What would have been needed to achieve that change?

What did you think of the proposed adaptations that were presented?

**After the DAP**

How has your contact with the rehabilitation coordinator been after the three-party meeting? What expectations did you have after the DAP regarding your work?

*Expectations of your manager*
*Adjustments*
*Expectations of rehabilitation*

How do you feel that the three-party meeting and what you discussed in the DAP influenced your return to work?
What has happened to the adjustments you and your manager agreed to?
Have your work situation/tasks changed since you took part in the DAP?

What is your work situation like now?
How much do you work?
Short-term absence?
What do your main tasks consist of?

Now, I have asked a lot of questions. Is there anything you feel you want to share, that has not been brought up, that feels especially important in this context?
